# COVID-19 classification using chest X-ray images based on fusion-assisted deep Bayesian optimization and Grad-CAM visualization

**DOI:** 10.3389/fpubh.2022.1046296

**Published:** 2022-11-04

**Authors:** Ameer Hamza, Muhammad Attique Khan, Shui-Hua Wang, Majed Alhaisoni, Meshal Alharbi, Hany S. Hussein, Hammam Alshazly, Ye Jin Kim, Jaehyuk Cha

**Affiliations:** ^1^Department of Computer Science, HITEC University, Taxila, Pakistan; ^2^Department of Mathematics, University of Leicester, Leicester, United Kingdom; ^3^Computer Sciences Department, College of Computer and Information Sciences, Princess Nourah Bint Abdulrahman University, Riyadh, Saudi Arabia; ^4^Department of Computer Science, College of Computer Engineering and Sciences, Prince Sattam Bin Abdulaziz University, Al-Kharj, Saudi Arabia; ^5^Electrical Engineering Department, College of Engineering, King Khalid University, Abha, Saudi Arabia; ^6^Electrical Engineering Department, Faculty of Engineering, Aswan University, Aswan, Egypt; ^7^Faculty of Computers and Information, South Valley University, Qena, Egypt; ^8^Department of Computer Science, Hanyang University, Seoul, South Korea

**Keywords:** corona virus, multi-filters contrast enhancement, deep learning, Bayesian optimization, hyperparameters, fusion

## Abstract

The COVID-19 virus's rapid global spread has caused millions of illnesses and deaths. As a result, it has disastrous consequences for people's lives, public health, and the global economy. Clinical studies have revealed a link between the severity of COVID-19 cases and the amount of virus present in infected people's lungs. Imaging techniques such as computed tomography (CT) and chest x-rays can detect COVID-19 (CXR). Manual inspection of these images is a difficult process, so computerized techniques are widely used. Deep convolutional neural networks (DCNNs) are a type of machine learning that is frequently used in computer vision applications, particularly in medical imaging, to detect and classify infected regions. These techniques can assist medical personnel in the detection of patients with COVID-19. In this article, a Bayesian optimized DCNN and explainable AI-based framework is proposed for the classification of COVID-19 from the chest X-ray images. The proposed method starts with a multi-filter contrast enhancement technique that increases the visibility of the infected part. Two pre-trained deep models, namely, EfficientNet-B0 and MobileNet-V2, are fine-tuned according to the target classes and then trained by employing Bayesian optimization (BO). Through BO, hyperparameters have been selected instead of static initialization. Features are extracted from the trained model and fused using a slicing-based serial fusion approach. The fused features are classified using machine learning classifiers for the final classification. Moreover, visualization is performed using a Grad-CAM that highlights the infected part in the image. Three publically available COVID-19 datasets are used for the experimental process to obtain improved accuracies of 98.8, 97.9, and 99.4%, respectively.

## Introduction

The coronavirus has recently spread throughout the world as a new infection. Coronavirus is typically spread by animals or humans (1, 2). It is discovered to be transmitted by bats as a result of animal transmission. Coronavirus also replicates in the human body with several other common coronaviruses, including 229E: Alpha, NL63: Alpha, OC43: Beta, and HKU1: Beta (3). The coronavirus disease outbreak was dubbed the coronavirus global pandemic or COVID-19 pandemic by the World Health Organization (WHO) in March 2020 (4). The disease known as COVID-19 is caused by a virus (SARS-CoV-2). Lung diseases range in severity from a common cold to a potentially fatal illness. Coronavirus illnesses were frequently accompanied by respiratory system diagnoses. Individuals may occasionally contract minor, self-limiting infections with severe consequences, such as influenza. Symptoms of respiratory problems, fatigue, and a sore throat include fever, cough, and breathing difficulties (5). The majority of researchers have emphasized the need for COVID-19-specific diagnostic methods, medications, or vaccinations to prevent its spread (6). Because of its higher sensitivity and specificity in terms of observations, the reverse transcription-polymerase chain reaction (RT-PCR) is the current gold standard for diagnosing COVID-19 (7).

Visual indicators could be used as an alternative strategy for quickly screening infected individuals (8). This infection's most prevalent symptom is respiratory sickness. For chest radiography, images (X-rays of the chest) are thought to be the most reliable visual signal. Radiologists examine these images physically to identify visual patterns that indicate the presence of COVID-19 (9). Even though traditional diagnosis has improved over time, it is still vulnerable to medical staff errors. It is also more expensive because each patient requires a diagnostic test kit. Medical-based imaging procedures, such as CXR and CT scans, are much faster, safer, and more widely available for screening (10). For COVID-19 screening, CXR image screening is superior to CT scans because it is more accessible and less expensive (11, 12). However, it may take some time to manually diagnose the virus using X-ray scans. If there is little or no prior knowledge and expertise about the infection and its characteristics, it may result in several inaccuracies and human-made mistakes. As a result, there is a compelling need to automate such operations on a large scale, and it should be accessible to all, so that treatment can become more effective, precise, and timely (13).

Previous research has used computer vision (CV) and artificial intelligence (AI) methods involving deep learning (DL) algorithms; specifically, CNNs have been validated as a realistic method for analyzing medical images (14, 15). A deep learning technique called a convolutional neural network was previously utilized to accurately identify pneumonia in CXR images of a patient's chest (16–18). The researchers introduced several CNN models for classification tasks, including ResNet50 (19), AlexNet (20), InceptionV3 (21), and a few others (22). Computer vision researchers have used pre-trained deep learning models in medical imaging, particularly for COVID-19 diagnosis and classification (23, 24).

Loey et al. (25) presented a Bayesian-based optimization DCNN model to classify coronavirus illness by using CXR images. The presented approach tuned the hyperparameters of DCNN models and extracted the high-level features. The data used in the experimental process were large in size and achieved 96% accuracy. This approach is limited by its high computing time due to the Bayesian optimization because it takes too much iteration during the training process. Yoo et al. (26) employed a hybrid technique model on CXR images by classifying the coronavirus using a decision tree classifier and deep learning. The created method achieved 95% accuracy. Wang et al. (27) designed a deep learning model-based transfer learning approach to identify the coronavirus. CXR images were utilized for this method. COVID-19 and healthy images were 565,537, respectively. The created deep learning technique gained 96.7% accuracy. They extracted high-level features and ML-based classifiers to create an efficient technique for improving the sensitivity of DCNN models. Chowdhury et al. (28) implemented a novel framework based on a CNN. They used a multiclass dataset that included COVID-19, pneumonia, and the healthy class. They constructed a CNN in the parallel pipeline and supplied crucial elements for the classification method. The suggested approach attained an accuracy of 96.9%, which was superior to the current techniques. Khan and Aslam (29) utilized the DCNN networks such as ResNet, DenseNet, and VGGNet and performed transfer learning concepts for training the models on the Chest X-ray dataset. The dataset includes 195 COVID-19 images and 862 normal images. On the selected dataset, the provided method achieved an accuracy of 99%. Che Azemin et al. (30) designed a ResNet CNN based on a deep learning algorithm to diagnose COVID-19 from CXR images. They considered the binary class problem—COVID-19 and healthy classes. The selected dataset was utilized for the training of the CNN model through transfer learning. The trained model achieves 72% accuracy, which is higher than the recent methods. Khan et al. (31) presented a DL and explainable AI-based framework for COVID-19 classification from CXR images. Transfer learning was utilized to train pre-trained deep models on enhanced images, and features were merged for greater information. Following that, the Whale–Elephant herding method is used to choose the best features, which are then classified using the ELM classifier. Few other techniques such as meta-classifier with deep learning approach for COVID-19 classification (32), novel CNN approach called CNN-COVID (33), optimization algorithm called novel crow swarm (34), and multi-agent deep reinforcement learning (35).

The models in the preceding studies were retrained using the transfer learning concept, which involves freezing the weights of a few layers to save computational time. They also used fixed hyperparameters like learning rate, momentum, mini-batch size, epoch count, etc. When there is a lot of variation in the results due to different hyperparameter values, this method is inefficient. In this work, we proposed a multimodal Bayesian hyperparameter optimization method for the training of deep learning models for COVID-19 classification. Moreover, an explainable AI-based diagnosis has been performed. Our major participation in this work is as follows:

- A multi-filter fusion-based hybrid technique is proposed for contrast enhancement that increases the local and global information of an image.
- Bayesian optimization is employed on deep learning models for the optimization of hyperparameters that helps in the better training of selected data.
- High-level features are extracted by both models and fused by a novel slicing-based serial fusion.
- Grad-CAM visualization is performed on the final classification, resulting in the colored visualization of the COVID-19, pneumonia, and tuberculosis-infected regions.

The manuscript is organized as follows. The proposed methodology such as multi-filters fusion-based hybrid contrast enhancement technique, Bayesian optimization of hyperparameters of DCCN models, deep transfer learning, feature extraction, fusion, and Grad-CAM for explainable AI, is presented in Section Proposed methodology. The findings of the proposed approach are shown in Section Experimental results and analysis, and Section Conclusion presents the conclusion.

## Proposed methodology

**Figure 1:**
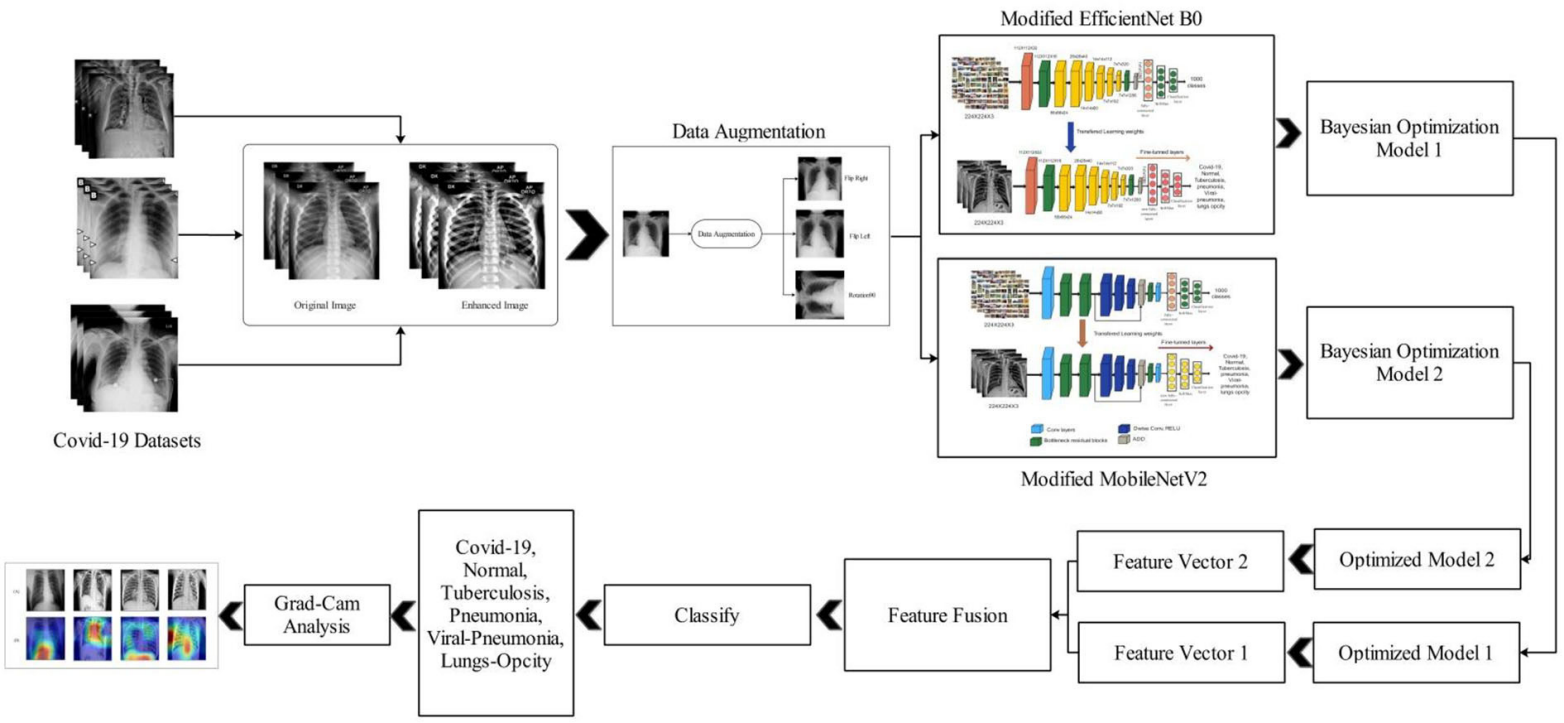
Proposed classification architecture for COVID-19 utilizing deep transfer learning and Bayesian optimization.

The proposed methodology for the COVID-19 classification and explainable AI-based diagnosis is presented here. In the proposed method, multi-filter contrast enhancement and deep transfer learning with Bayesian optimization are employed. Figure 1 shows the proposed architecture based on Bayesian optimization and features fusion for COVID-19 classification. This figure illustrates that, in the first phase, data augmentation is performed on the selected datasets using a multi-filter contrast enhancement method and a few additional filters. Two pre-trained models, namely, EfficientNet-B0 and MobileNet-V2, are modified and trained using deep transfer learning and optimized the hyperparameters by employing Bayesian optimization. Features are extracted from both optimized models and fusion is performed by utilizing a new method named, slicing-based serial fusion. Finally, the samples are subjected to Grad-CAM analysis in order to pinpoint the source of the infection.

### Contrast enhancement

Enhancing contrast is one of the most important and useful steps to enhance the vital objects in the images (36). Another goal of this step is to enhance the overall image quality. Medical image identification and interpretation mainly rely on image enhancement methods (37). When segmentation is performed, poor contrast is always detected incorrectly. In the classification process, the enhanced images can extract more important features than the feature extraction through the original images. In this work, the images of the selected datasets have low contrast and poor quality. These problems may lead us to misclassification. Therefore, we designed a multi-filter technique by utilizing the fusion of different filters. First, top-hat and bottom-hat filtering are implemented and combined with information. After that, intensity values are adjusted by using a mathematical formula.

**Figure 2:**
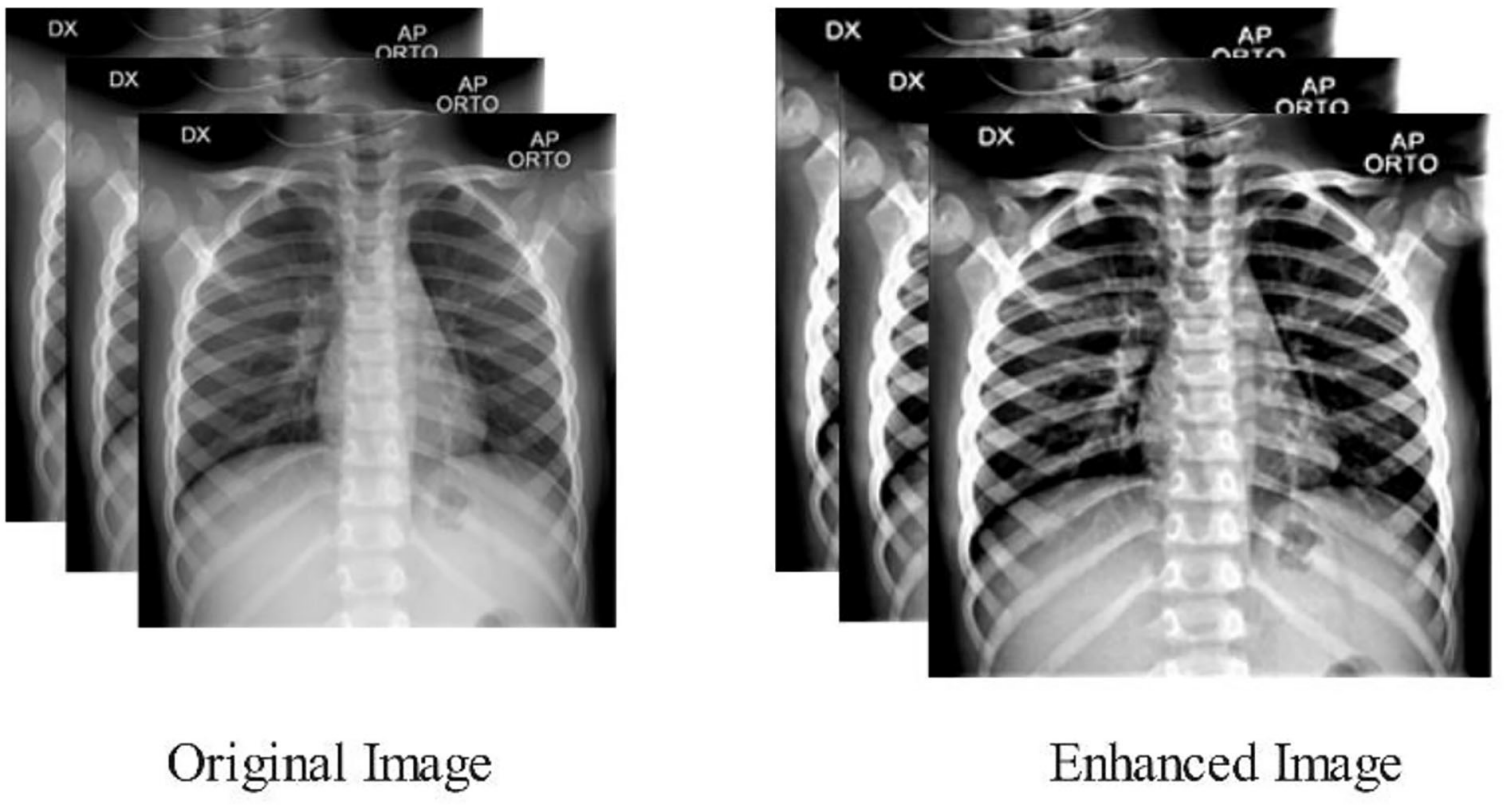
Samples enhanced images of multi-filters fusion technique.

Consider, the COVID-19 datasets 𝔻 having *k* images 𝔻∈ℝ^*k*^, where each image represented by $T^{k}(h,w)$ and (*h, w*) ∈ ℝ. Each sample has resized into N×M=224. Suppose that the kernel σ with a value of 13. The top-hat is based on • opening operation and the bottom-hat is based on ▪ closing operation. So, the top-hat and bottom-hat filtering are derived as:


(1) $$\begin{array}{lll} T_{top}\left(h,w\right) & = & T^{k}\left(h,w\right)-\left(k^{k}\left(h,w\right)\;{\;}^{◦}\;\sigma \;\right) \end{array}$$

(2) $$\begin{array}{lll} T_{bottom}\left(h,w\right) & = & \left(k^{k}\left(h,w\right)\;▪\;\sigma \;\right)-T^{n}\left(h,w\right) \end{array}$$

(3) $$\begin{array}{lll} f^{\prime }\left(h,w\right) & = & T^{n}\left(h,w\right)+T_{top}\left(h,w\right)-T_{bottom}\left(h,w\right) \end{array}$$

where *f*′(*h, w*) represents the fused image of top-bottom filtering. In the next step, the adjust filter is employed on the resultant images from the top-hat and bottom-hat filters. Adjust filter boosts an image's lightness by transforming the points of the input pixels' intensities to new ones, with the mean amount of data absorbed in the low and high intensities being about 1.5%. The symbol *p* is the pixel value of the image, the *gamma (*γ*)* is a variable, which evaluates the form of the procedure among the coordinating coefficients (*q, f*) and (*r, e*).


(4) $$\begin{array}{lll} {Adj}^{k}\left(h,w\right) & = & {\left(\frac{p-q}{r-q}\right)}^{\gamma }\left(e-f\right)+e \end{array}$$

(5) $$\begin{array}{lll} F^{k}\left(h,w\right) & = & f^{\prime }\left(h,w\right)+{Adj}^{k}\left(h,w\right) \end{array}$$

where *F*^*k*^(*h, w*) is the final enhanced image, visually illustrated in Figure 2.

### Dataset collection and description

**Figure 3:**
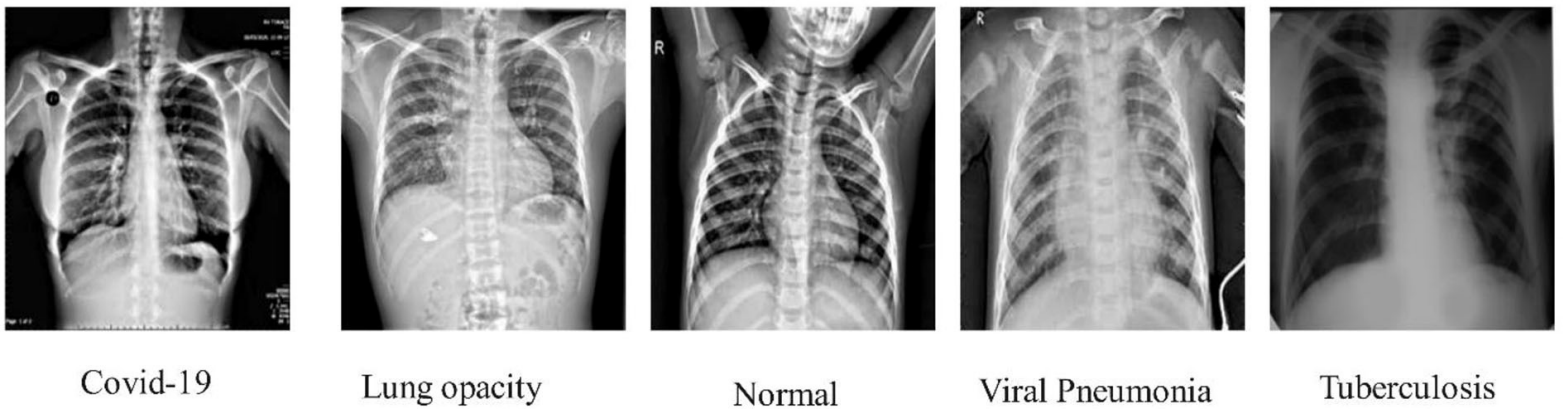
sCXR instances for the classification of COVID-19 and other infections.

**Figure 4:**
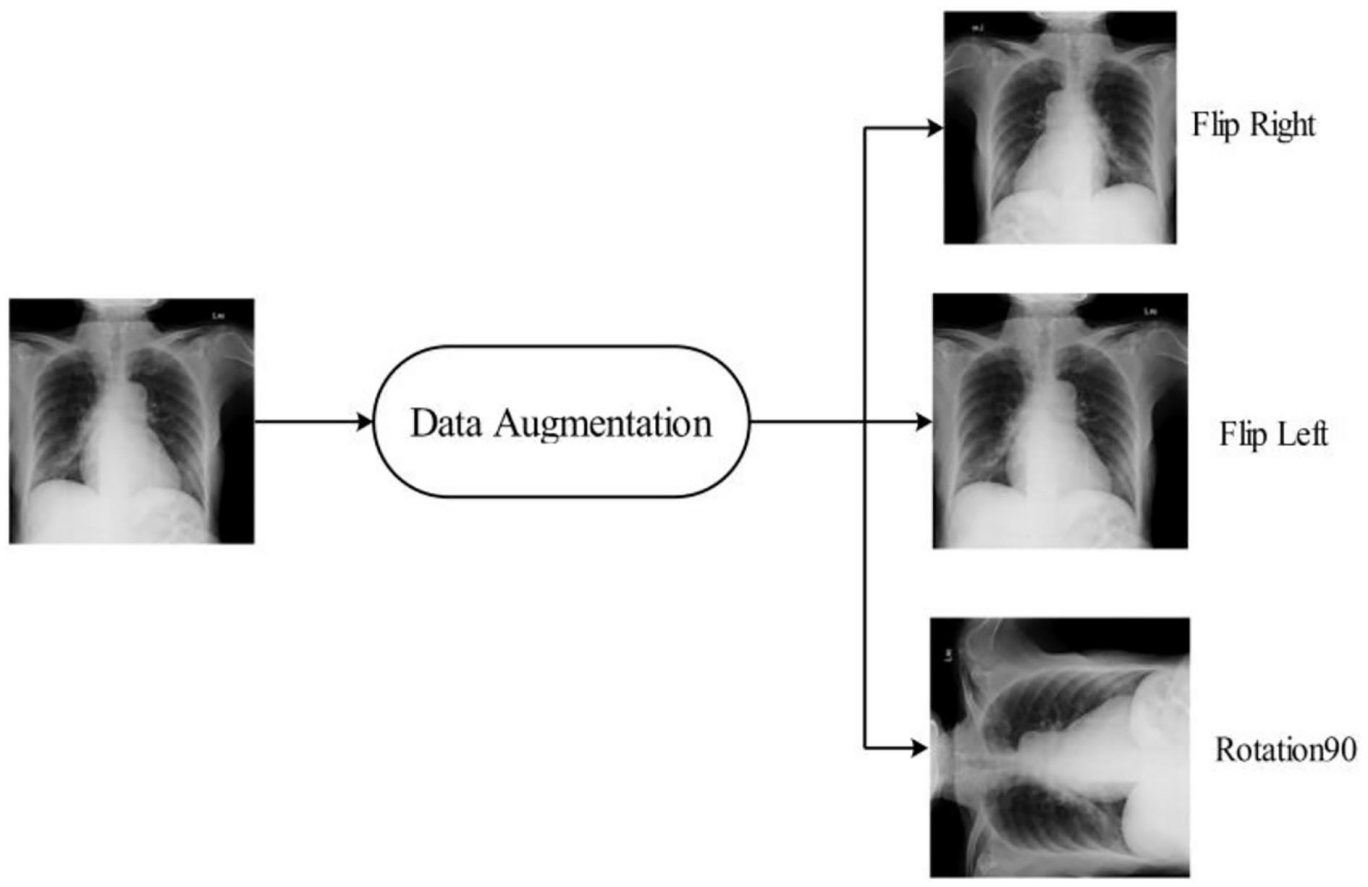
Sample images after data augmentation.

This study adopts an experimental technique that makes use of three publicly accessible datasets: COVID-GAN and COVID-Net small chest x-ray (https://www.kaggle.com/yash612/covidnet-mini-and-gan-enerated-chest-xray), COVID-19 radiography (https://www.kaggle.com/datasets/tawsifurrahman/covid19-radiography-database). CXR (pneumonia, COVID-19, TB) (https://www.kaggle.com/datasets/jtiptj/chest-xray-pneumoniacovid19tuberculosis). There are three classes in COVID-GAN and COVID-Net small chest x-ray datasets. COVID-19 radiography and CXR (pneumonia, COVID-19, and tuberculosis) consist of four classes. The original images are shown in Figure 3. These datasets are highly imbalanced as shown in Table 1. For balancing the dataset, we set 6,000 images in each class for all the datasets by utilizing data augmentation. Using the augmented dataset, 50% of images have been utilized for the training, while the rest of the 50% were used for the testing. In the data augmentation process, three primary functions are used: flip-left, rotate 90, and flip-right. The augmented images are visually shown in Figure 4.

**Table 1:** Complete explanation of selected datasets.

**Classes**	**Original images**	**Augmented images**	**Training/testing images**
**COVID-19 radiography database**			
COVID-19	3,616	6,000	3,000/3,000
Lung opacity	6,012	6,000	3,000/3,000
Normal	10,192	6,000	3,000/3,000
Viral pneumonia	1,345	6,000	3,000/3,000
**COVID-GAN and COVID-Net mini–Chest X-ray**			
Corona	461	6,000	3,000/3,000
Normal	1,575	6,000	3,000/3,000
Pneumonia	4,481	6,000	3,000/3,000
**CXR (pneumonia, COVID-19, tuberculosis)**			
COVID-19	566	6,000	3,000/3,000
Normal	1,575	6,000	3,000/3,000
Pneumonia	4,265	6,000	3,000/3,000
Tuberculosis	491	6,000	3,000/3,000

### EfficientNet deep features

The EfficientNet model, which ranks among the top models, achieved 84.4% accuracy on ImageNet for the classification task with a parameter size of 5.3 M (38). Deep learning architectures are intended to find simple but efficient solutions. By uniformly increasing depth, breadth, and resolution while reducing model size, EfficientNet outperforms competitor state-of-the-art models. The first step in the compound scaling technique is to find a grid that identifies whether distinct scaling dimensions of the baseline network connect to one another within the limits of a constrained set of resources. The optimal scaling factor for height, breadth, and resolution may be determined using this procedure. These coefficients are then added to the original network to make the final network the appropriate size (17).

**Figure 5:**
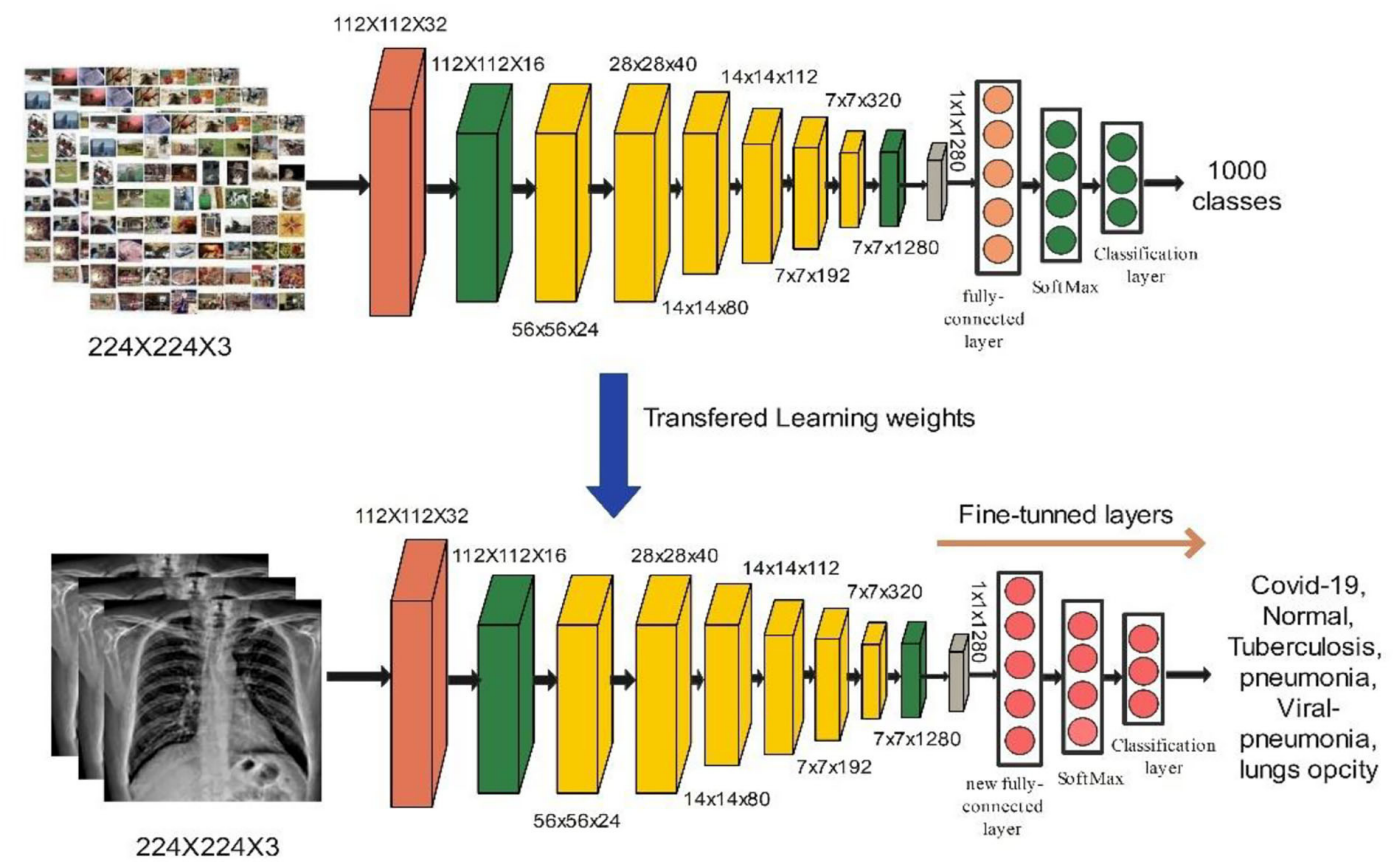
Visually representation of modified building block of efficient Net b0.

The main building block for EfficientNet-B0 is the asymmetrical bottleneck MB Conv. Blocks in MB Conv consist of an expansion layer followed by a compression layer. Later, it was possible to connect bottlenecks directly while connecting a much smaller number of channels. When compared to conventional layers, the computational cost of this design's deep separable convolutions is around *k*^2^, where k is the kernel size that determines the width and height of the 2D convolution window (39). In this work, we utilized the EfficientNet-B0 model for the features extraction. The model was originally trained on 1,000 classes and accepts the input size of 224 × 224 × 3. We fine-tuned the FC Layer with the new FC Layer which consists of COVID-19 classes. The updated model was trained by utilizing deep transfer learning and BO. The detail of Bayesian optimization (BO) is provided below. The objective of BO was to find the best hyperparameters for EfficientNet-B0 which gives the minimum error rate and increase the accuracy. The hyperparameters are selected dynamically *via* BO. The high-level features extracted from the average global pooling layer after the model has been trained on selected COVID-19 datasets and obtained a feature vector of size *N* × 1,280. Visually, the process of fine-tuning and deep transfer learning is shown in Figure 5.

### MobileNet-V2 deep features

**Figure 6:**
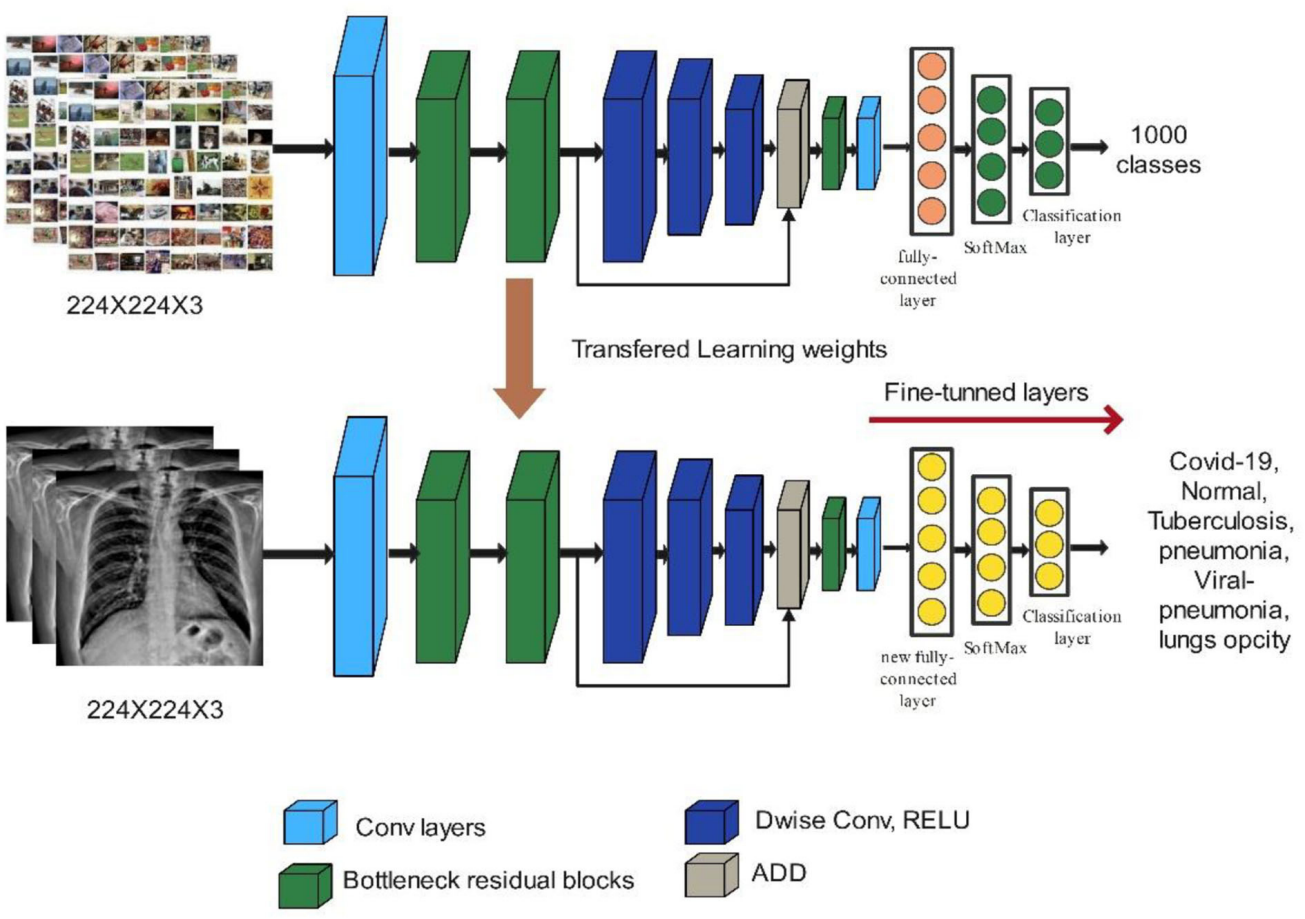
Modified building block of MobileNet-V2.

MobileNet-V2 employs depth-wise separable convolutions (DSCs) for portability and to solve the problem of data loss in non-linear layers inside convolution blocks. MobileNet-V2 has 5.3 million parameter values (40). The building block of MobileNet-V2 is shown in Figure 6. We used the MobileNet-V2 model in our proposed work for deep feature extraction. The model was pre-trained on the ImageNet dataset, which has 1,000 classes, and it takes input sizes of 224 × 224 × 3. The FC Layer was replaced with a new FC layer. As described in Section Hyperparameters optimization using BO, the updated model was trained using deep transfer learning, and the hyperparameters were optimized using BO. The trained model was utilized for the feature extraction. The activation is performed on global average pool (GAP) layer and retrieved features have a dimension of *N* × 1280. Visually, the process of deep transfer learning is shown in Figure 6.

### Hyperparameters optimization using BO

When using deep learning architectures, we need to adjust all of the hyperparameters in order to obtain classification accuracy. The selection of hyperparameters has a significant impact on the accuracy of the correct prediction (41). The goal of optimizing hyperparameters is to choose the values that get the best validation results. The hyperparameter optimization is calculated as:


(6) $$\begin{array}{l} x^{*}=argmin\;f\left(x\right) \end{array}$$

where *f*(*x*) is the objective score to minimize error rate when compared to the validation set, and *x* is the set of hyperparameters with a value in the domain where hyperparameter optimization evaluation is more expensive. It takes longer to train and is nearly impossible to achieve by hand with deep neural network models with many hyperparameters. BO has been used in simulations and machine learning models. To improve model performance, computer vision-based approaches use feed-forward network architectures to adjust hyperparameters. It simplifies the time-taking task of optimizing a number of parameters (42). Deep learning models need particular hyperparameter (HP) tuning. These parameters can be manually or automatically set. Although manual optimization produces adequate results, it is highly dependent on expertise and lacks consistency, making it less than ideal. HPs can be modified automatically by random and grid searches; however, some ineffectual sites may be unavoidable due to the inability to gain knowledge from previous searches. BO has garnered a lot of attention in parameter modification because of its distinct advantages. BO differs from other approaches in that it takes into account historical parameter information by updating the prior with Gaussian progress (GP). Also, BO has a very low number of iterations and a very fast convergence time. The BO method may also avoid local optimality when dealing with non-convex situations. The strong convergence and robustness of BO make it an excellent choice for optimizing HPs (43, 44). In our work, we utilized Bayesian optimization for a deep convolutional neural network to optimize the hyperparameters for achieving the minimum error of models. Section Depth, learning rate, momentum, and L2Regularization are the optimization parameters. The ranges of these parameters are shown in Table 2.

**Table 2:** Hyperparameters ranges for Bayesian optimization.

**Hyperparameters**	**Ranges**
Section depth	(1, 3)
Learning rate	[0.001, 1]
Momentum	[0.8, 0.98]
L2Regularization	[1*e*^−10^, 1*e*^−2^]

### Proposed feature fusion

Feature fusion is an important step in which multi-directional information is combined to get a better output. As shown in Figure 1, features are extracted from two pre-trained models; therefore, fusion is important to combine the only important information (36). We proposed a novel feature fusion technique called slicing-based serial fusion in our study.

**Figure 7:**
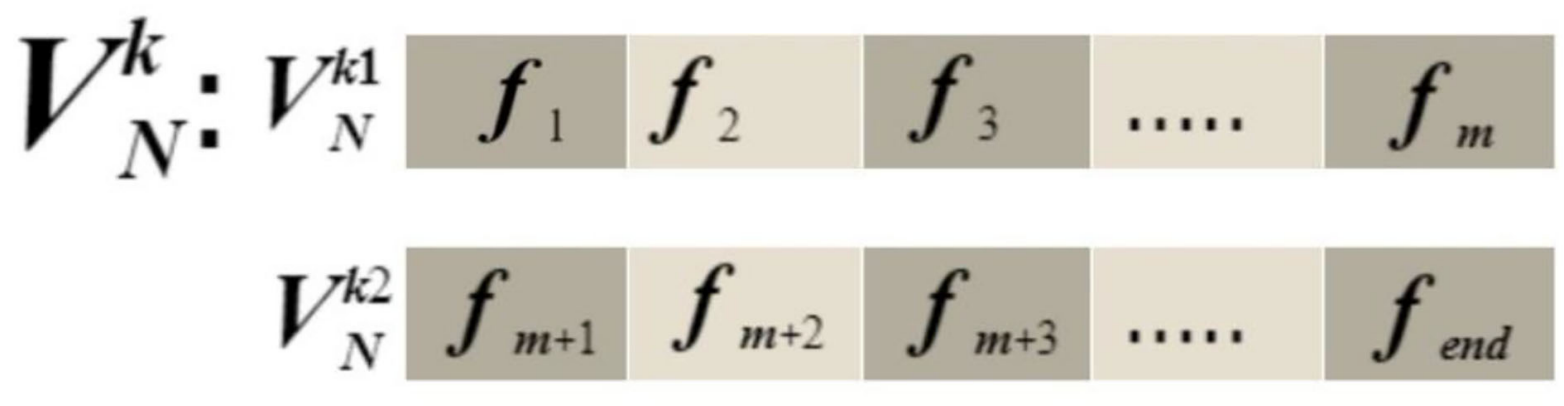
Structure of slicing vectors.

Consider, the first vector $V_{N}^{k1}$, which has a dimension of *N* × 1280, and the size of second vector $V_{N}^{k2}$, which also has a dimension of *N* × 1280 and is obtained by selected models EfficientNet-B0 and MobileNet-V2, respectively. Suppose $V_{N}^{k3}$ is fused feature vector having dimension *N* × ***K***. We selected a mid-point based on any from the selected vectors, which are computed as follows:


(7) $$\begin{array}{l} m=\frac{N}{2} \end{array}$$

where *m* represents the mid-point of vector and *N* represents the total number of images used for feature extraction. Based on the *m*-value, $V_{N}^{k1}\;$and $V_{N}^{k2}$ are divided into slices. The slices equation is calculated as:


(8) $$\begin{array}{lll} V_{N}^{11} & = & V_{N}^{k1}\left(f_{1,}f_{2,}f_{3}\ldots f_{m}\right) \end{array}$$

(9) $$\begin{array}{lll} V_{N}^{12} & = & V_{N}^{k1}\left(f_{m+1}\ldots f_{end}\right) \end{array}$$

(10) $$\begin{array}{lll} V_{N}^{21} & = & V_{N}^{k2}\left(f_{1,}f_{2,}f_{3}\ldots f_{m}\right) \end{array}$$

(11) $$\begin{array}{lll} V_{N}^{22} & = & V_{N}^{k2}\left(f_{m+1}\ldots f_{end}\right) \end{array}$$

where $V_{N}^{11}$ and $V_{N}^{12}$ represent the slicing that contains half and half of the features of vector $V_{N}^{k1}$, and $V_{N}^{21}$ and $V_{N}^{22}$ represent the slicing that contains half and half of the features of vector $V_{N}^{k2}$. The structure of slicing vectors is visually shown in Figure 7.

After slicing both vectors, the information is aggregated in the initial fused vector $V_{fused}$. The sliced vectors are fused in this sequence $V_{N}^{11},V_{N}^{21},\;V_{N}^{12},V_{N}^{22}$, respectively.


(12) $$\begin{array}{l} V_{fused}={\left(\begin{array}{c} V_{N}^{11}\\ V_{N}^{21}\\ \;V_{N}^{12}\\ V_{N}^{22} \end{array}\right)}_{K\times N} \end{array}$$

The output vector $V_{fused}$ are attained with dimensions *N* × 2560 but these features are mixed with each other by utilizing the slicing technique. In the next phase, features are refined further using a Kurtosis-based function. We tried to select the important features in the fused vector using this function.


(13) $$\begin{array}{lll} Kr & = & \frac{\mu_{4}}{\sigma^{4}} \end{array}$$

(14) $$\begin{array}{lll} \mu_{4} & = & \frac{E\left[{\left(V-\mu \right)}^{4}\right]}{\sigma^{4}} \end{array}$$

(15) $$\begin{array}{lll} \sigma^{2} & = & \sqrt{E\left[{\left(V-\mu \right)}^{2}\right]},\sigma =\sqrt{\sigma^{2}} \end{array}$$

(16) $$Fusion\;=\{\begin{array}{c} V_{N}^{k3}\;for\;V_{fused}\geq Kr\;\\ Ignore,\;Elsewhere \end{array}$$

Based on this equation, we obtained a final vector having dimension *N* × 1422. This resultant vector is fed to machine learning classifiers for final classification.

## Experimental results and analysis

For the experimental process, the datasets are split 50:50, indicating that 50% of the images are used to train the models and the remaining 50% are utilized for the testing process. The entire experimental process is carried out using 10-fold cross-validation. The static hyperparameters that are used during the training of deep models are epochs and mini-batch sizes having values 200 and 16, respectively. Moreover, the initial learning rate, stochastic gradient descent, momentum, L2Regularization, and section depth are optimized by utilizing Bayesian optimization. Multiple classifiers are used in this work for the classification results, including a support vector machine, wide neural network, ensemble subspace discriminant, and linear regression kernel. The classifier's performance parameters are sensitivity, precision, false positive rate, F1-score, accuracy, and computation time. Moreover, Grad-CAM analysis is conducted for further verification of the infected COVID-19 region in the image. All the simulations are conducted in MATLAB2022a executing on a workstation from MSI's GL75 Leopard series equipped with an 8 GB NVIDIA GTX graphics card, 512 SSD, and an Intel Core i7 10th generation processor.

### COVID-19 radiography database results

#### Modified EfficientNet-B0 features

In this experiment, features are extracted from modified EfficientNet-B0. This model was trained through BO and transfer learning on the augmented dataset. Table 3 shows the classification accuracy of this updated model on the COVID-19 radiography dataset. In this table, it is noted that the QSVM classifier has a higher accuracy of 97.2% than the other classifiers listed. This classifier has a sensitivity rate of 97.12%, a precision rate of 97.15%, and an F1-score of 97.13%. Additionally, these values are determined for the remaining classifiers. During the classification process, the computation time of all classifiers is also recorded, with the wide neural network consuming the least time 18.004 (s) and the SVM kernel classifier taking the most time (688.63) (s).

**Table 3:** Classification accuracy of modified EfficientNet-B0 Bayesian optimization features on COVID-19 radiography dataset.

**Classifiers**	**Sensitivity**	**Precision**	**FPR**	**F1-score**	**Accuracy**	**Time**
**QSVM**	**97.12**	**97.15**	**0.0075**	**97.13**	**97.2**	**19.996**
CSVM	97.22	97.22	0.0075	97.22	97.2	21.042
M G SVM	97.02	97.05	0.01	97.03	97.0	21.943
C G SVM	96.70	96.75	0.01	96.72	96.08	21.283
ESD	96.75	96.77	0.01	96.75	96.9	82.864
LSVM	97.05	97.07	0.10	97.05	97.0	18.952
SVM Kernel	96.92	96.95	0.0075	96.93	96.9	688.63
LRK	96.55	96.57	0.035	96.55	96.5	212.31
LD	96.55	96.57	0.010	96.55	96.5	18.935
WNN	95.52	96.57	0.015	96.04	96.5	18.004

Bold represent best values.

#### Modified MobileNet-V2 features

From this experiment, the modified MobileNet-V2 model is fine-tuned and trained using BO on the COVID-19 radiography dataset. Table 4 shows the classification results of this experiment. From this table, the ESD classifier has an accuracy of 94.2% that is better than the other classifiers, listed in this table. This classifier has a 94.25% sensitivity rate, 94.28% precision rate, and F1-score is 94.28%. The numerical outcomes support the conclusion that the ESD outperforms the other classifiers. These values are also generated for the experiment's remaining classifiers. In this experiment, the amount of time for each classifier is noted and the linear discriminant classifier required the least amount of time of 15.743 s. In contrast, the LRK classifier was executed in 127.41 s, the highest of all the classifiers.

**Table 4:** Classification accuracy of modified MobileNet-v2 Bayesian optimization features on COVID-19 radiography dataset.

**Classifiers**	**Sensitivity**	**Precision**	**FPR**	**F1-score**	**Accuracy**	**Time**
QSVM	92.85	92.92	0.022	92.88	92.8	28.351
CSVM	92.20	92.27	0.025	92.23	92.2	33.41
M G SVM	91.05	91.20	0.027	91.12	91.0	33.386
C G SVM	87.52	88.90	0.04	88.20	87.5	50.183
**ESD**	**94.25**	**94.32**	**0.02**	**94.28**	**94.2**	**106.56**
LSVM	93.75	93.80	0.022	93.77	93.8	20.353
SVM Kernel	94.25	94.32	0.017	94.28	94.2	89.6
LRK	90.45	90.62	0.032	90.53	90.8	127.41
LD	83.92	87.05	0.052	85.45	83.9	15.743
WNN	91.37	91.42	0.027	91.39	91.5	42.71

Bold represent best values.

#### Proposed fused results

**Figure 8:**
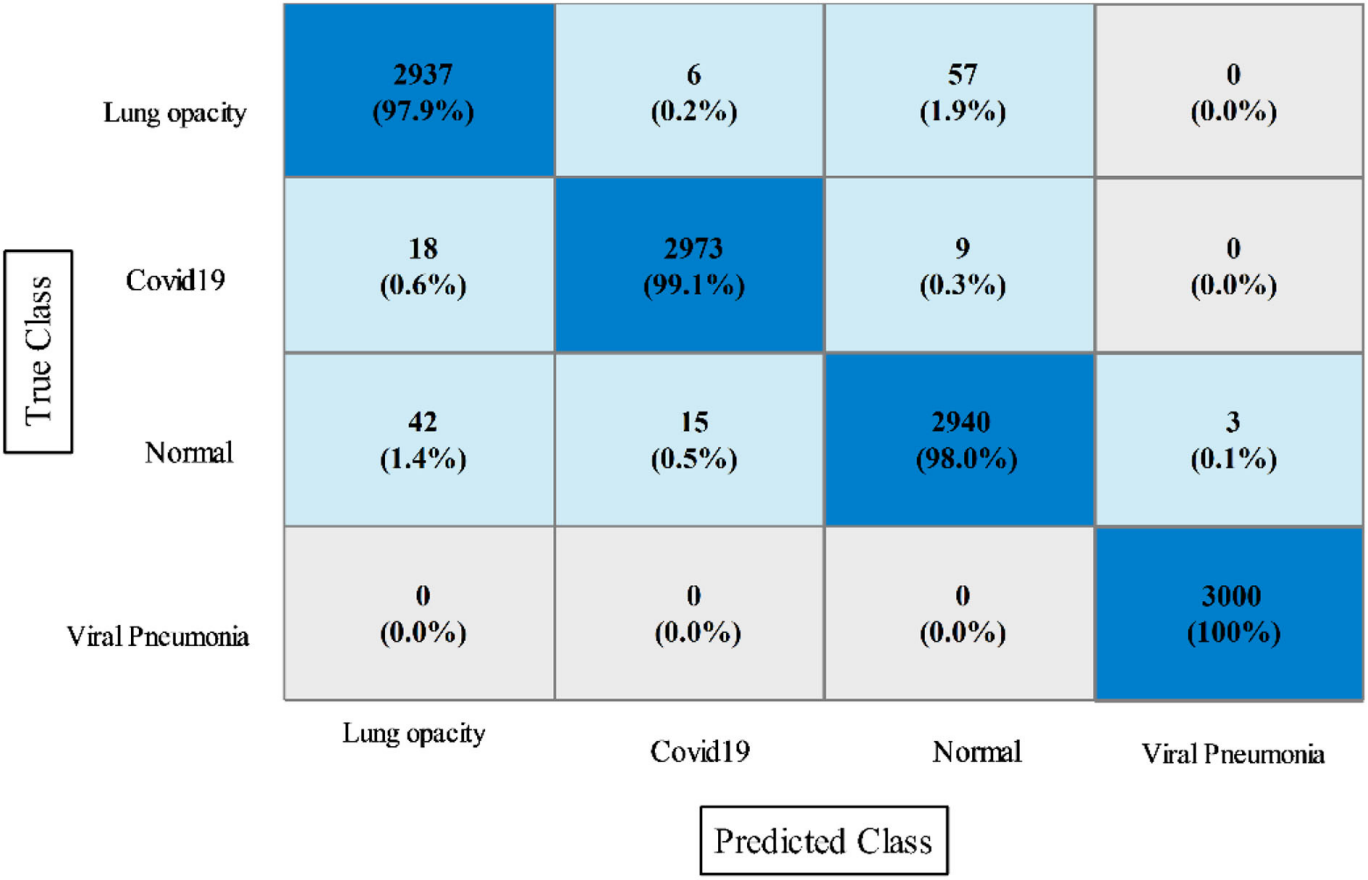
Confusion matrix of quadratic SVM utilizing the proposed slicing-based serial fusion technique on the COVID-19 radiography dataset.

In this experiment, the proposed fusion approach is opted and fused the features of both optimized models. The fused vector is passed to the classifiers, which yielded the best accuracy of 98.8% on QSVM, which is higher than in experiments 1 and 2. Table 5 shows the detailed results of this experiment. QSVM has a sensitivity rate of 98.57%, a precision rate of 98.57%, and an F1-score of 98.57%. In addition, a QSVM confusion matrix is shown in Figure 8. This statistic indicates that the correct prediction rate for each class exceeds 97%. Also observed is the computing time of each classifier, with the linear SVM classifier executing faster than the others. This classifier's execution time is 38.901 s, while the longest execution time is 454.86 seconds (s). Comparing Tables 3, 4, it is observed that the fusion process improves accuracy, but time is increased due to the addition of extra features.

**Table 5:** Classification results of proposed slicing-based serial fusion technique on COVID-19 radiography database.

**Classifiers**	**Sensitivity**	**Precision**	**FPR**	**F1-score**	**Accuracy**	**Time**
**QSVM**	**98.57**	**98.75**	**0.004**	**98.75**	**98.8**	**42.023**
CSVM	98.56	98.68	0.004	98.68	98.7	44.317
M G SVM	97.14	97.46	0.008	97.43	97.4	52.454
C G SVM	97.32	97.60	0.008	97.60	97.6	69.348
ED	98.15	98.45	0.005	98.47	98.5	454.86
LSVM	98.25	98.48	0.005	98.42	98.5	38.901
SVM Kernel	98.35	98.56	0.006	98.51	98.6	95.00
LRK	97.69	97.73	0.007	97.88	97.9	147.65
LD	94.56	94.62	0.017	97.65	94.7	42.13
WNN	97.82	97.90	0.006	98.07	98.1	48.073

Bold represent best values.

### COVID-GAN and COVID-Net mini chest X-ray dataset

In this section, the results of the COVID-GAN and COVID-Net Mini Chest X-Ray dataset have been presented. In the first phase, features are extracted from the modified EfficientNet-B0 model. This model was trained through BO and transfer learning on the augmented dataset. Table 6 shows the classification accuracy of this model and obtained the 97.4% on Cubic SVM. The sensitivity, precision, and F1-score are 97.13, 97.26, and 97.15%, respectively. The classification computational time for all classifiers in this phase experiment is also recorded; the Quadratic SVM classifier has the shortest execution time of 14.318 (s) and the longest execution time of 128.74 (s). In the next phase, features are extracted through the modified MobileNet-V2 model. Features are passed to the classifiers and obtained the maximum accuracy of 93.5% on Cubic SVM, as shown in Table 7. This table shows that the sensitivity rate for Cubic SVM is 93.35%, the precision rate is 93.46%, and the F1-score is also 93.47%. All classifiers' processing times are also noted down, and it is noted that modified EfficientNet-Bo features work better than modified MobileNet-V2 features.

**Table 6:** Proposed modified EfficientNet-B0 Bayesian optimized features results on COVID-GAN and COVID-Net mini chest X-ray dataset.

**Classifiers**	**Sensitivity**	**Precision**	**FPR**	**F1-score**	**Accuracy**	**Time**
QSVM	97.13	97.16	0.01	97.14	97.1	14.318
**CSVM**	**97.13**	**97.26**	**0.01**	**97.15**	**97.4**	**16.054**
M G SVM	96.95	96.96	0.01	96.95	96.9	18.33
C G SVM	95.36	95.53	0.02	95.44	95.4	18.085
ESD	96.70	96.73	0.01	96.71	96.7	121.690
LSVM	96.50	96.56	0.01	96.52	96.5	15.486
SVM Kernel	96.60	96.66	0.01	96.62	96.6	347.63
LRK	96.00	96.06	0.01	96.02	96.0	128.74
LD	95.30	95.36	0.02	95.32	95.0	15.587
WNN	96.76	96.76	0.01	96.76	96.8	59.033

Bold represent best values.

**Table 7:** Proposed method modified MobileNet-V2 utilizing Bayesian optimization results on COVID-GAN and COVID-Net mini chest X-ray dataset.

**Classifiers**	**Sensitivity**	**Precision**	**FPR**	**F1-score**	**Accuracy**	**Time**
QSVM	93.43	93.44	0.032	93.37	93.4	21.75
**CSVM**	**93.35**	**93.46**	**0.032**	**93.47**	**93.5**	**19.246**
M G SVM	92.25	92.54	0.037	92.43	92.5	23.361
C G SVM	91.30	76.26	0.060	79.77	91.3	17.924
ESD	93.03	92.13	0.034	92.94	92.9	148.03
LSVM	93.16	93.24	0.034	93.09	93.1	15.974
SVM Kernel	92.37	92.41	0.038	92.28	92.4	396.83
LRK	91.82	91.83	0.040	91.73	91.7	140.18
LD	86.20	86.46	0.069	85.98	86.2	24.760
WNN	92.52	92.68	0.036	92.66	92.7	95.177

Bold represent best values.

**Figure 9:**
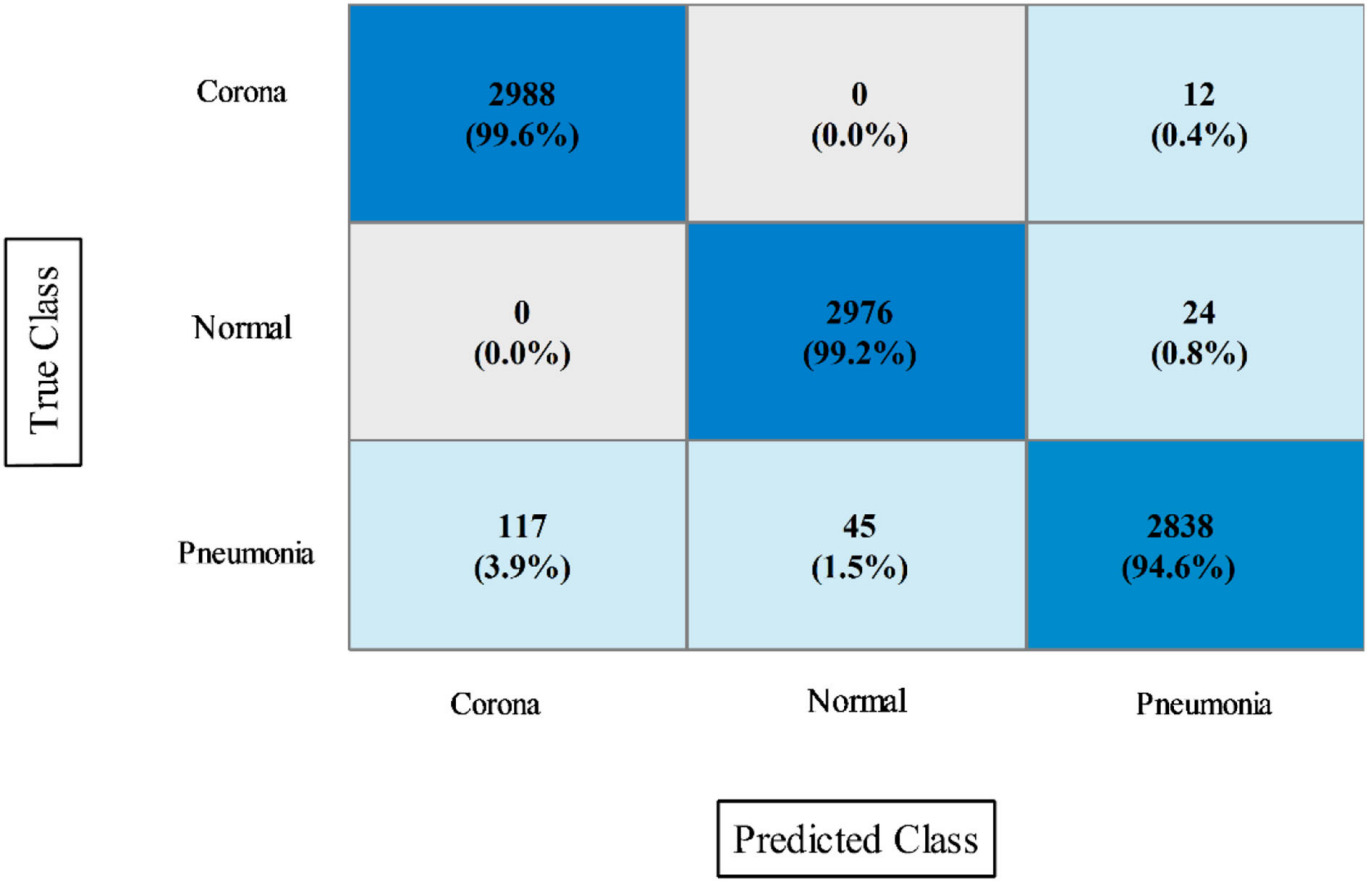
Confusion matrix of CSVM after proposed features fusion for COVID-GAN and COVID-Net mini chest X-ray dataset.

In the final step, fusion is performed using the proposed approach, and results are shown in Table 8. According to the data in this table, the Cubic SVM classifier has the highest accuracy of 97.9%, which is higher than the previous two steps (Tables 6, 7). The sensitivity and precision rates are also improved−97.26 and 97.36%, respectively. A confusion matrix, as shown in Figure 9, can be used to confirm the performance of CSVM. In comparison to the previous two experiments on this dataset, accuracy improves significantly after the fusion of features of both optimized trained models. Also, it is noted that the time is increased after the proposed fusion step.

**Table 8:** Proposed slicing-based serial fusion results on COVID-GAN and COVID-Net mini chest X-ray dataset.

**Classifiers**	**Sensitivity**	**Precision**	**FPR**	**F1-score**	**Accuracy**	**Time**
QSVM	97.42	97.56	0.012	97.59	97.6	36.804
**CSVM**	**97.26**	**97.36**	**0.010**	**97.89**	**97.9**	**39.492**
M G SVM	96.99	97.08	0.014	97.03	97.0	48.462
C G SVM	94.98	95.01	0.016	95.03	95.5	43.458
ESD	97.12	97.46	0.012	97.01	97.5	339.12
LSVM	96.91	96.97	0.013	97.00	97.1	34.862
SVM Kernel	96.95	96.99	0.012	97.02	97.3	432.8
LRK	95.97	96.01	0.015	96.04	96.5	169.25
LD	93.01	93.06	0.019	93.05	93.9	87.107
WNN	96.96	97.04	0.011	97.04	97.6	37.795

Bold represent best values.

### Chest X-ray (pneumonia, COVID-19, and tuberculosis)

**Figure 10:**
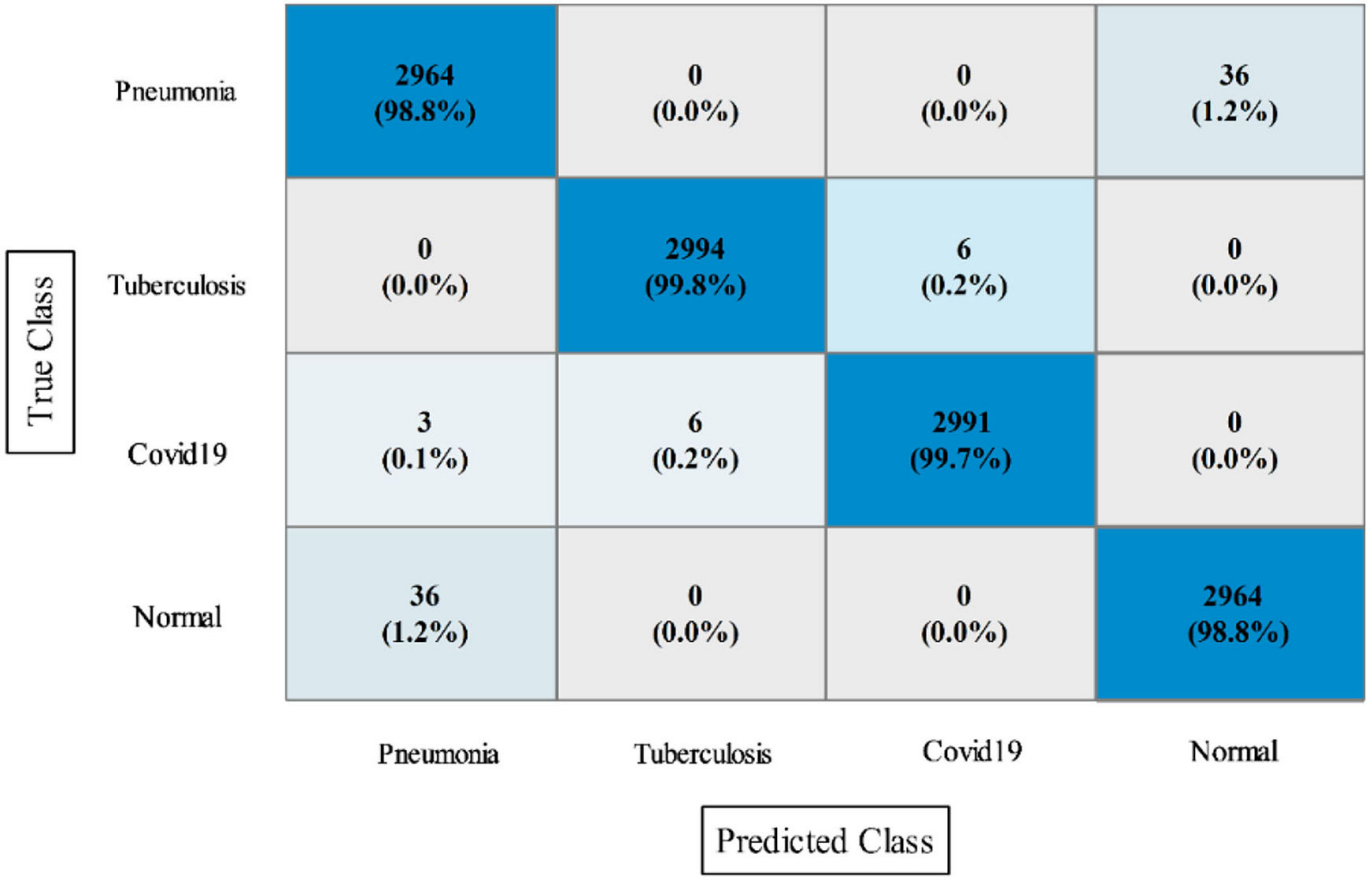
Confusion matrix of WNN for the proposed fusion on the chest X-ray dataset.

The results of this dataset are shown in Tables 9–11. Table 9 shows the results after the feature extraction through a modified EfficientNet-B0 model that was trained through BO. For these features, quadratic SVM gives a better accuracy of 98.80%. The linear SVM has the least execution time of 21.394 (s), whereas the SVM Kernel classifier has the highest execution time is 3,497.8 (s). Table 10 shows the classification results of modified MobileNet-V2 features. In this table, the QSVM obtained the best accuracy of 97.0%. The F1-score is 97.05, the sensitivity rate is 96.02, and the precision rate is 96.6%. The computational time of this model is a little high than the modified Efficientnet-B0 features. Finally, fusion is improved, and the outcomes are shown in Table 11. From this table, the wide neural network classifier has the highest accuracy of 99.4%. Other measures of this classifier are calculated as well, including an F1-score of 99.38%, a sensitivity rate of 99.63%, and a precision rate of 99.79%. The confusion matrix of this classifier is also shown in Figure 10. Based on this figure, the sensitivity rate can be verified. After the fusion process, computational time increases but significantly improves accuracy.

**Table 9:** Proposed modified EfficientNet-B0 Bayesian optimized features results on chest X-ray dataset.

**Classifiers**	**Sensitivity**	**Precision**	**FPR**	**F1-score**	**Accuracy**	**Time**
**QSVM**	**98.65**	**98.77**	**0.005**	**98.77**	**98.80**	**25.560**
CSVM	97.45	97.67	0.006	97.67	97.99	24.728
M G SVM	97.24	97.67	0.006	97.67	97.70	34.803
C G SVM	98.49	95.57	0.005	97.04	98.01	28.913
ESD	98.15	98.67	0.005	98.67	98.07	149.33
LSVM	98.14	98.72	0.005	98.72	98.70	21.394
SVM Kernel	98.65	98.73	0.005	98.05	98.07	3,497.8
LRK	98.23	98.32	0.005	98.32	98.30	290.05
LD	98.35	98.57	0.005	98.57	98.60	22.809
WNN	96.30	96.45	0.016	96.60	96.60	22.083

Bold represent best values.

**Table 10:** Proposed modified MobileNet-V2 Bayesian optimized features results on chest X-ray dataset.

**Classifiers**	**Sensitivity**	**Precision**	**FPR**	**F1-score**	**Accuracy**	**Time**
**QSVM**	**96.02**	**96.6**	**0.009**	**96.05**	**97.0**	**23.671**
CSVM	96.52	96.68	0.011	96.67	96.7	38.324
M G SVM	95.42	95.85	0.013	95.84	95.9	41.99
C G SVM	96.35	96.45	0.011	96.45	96.5	36.792
ESD	96.72	96.81	0.010	96.85	96.9	114.75
LSVM	96.58	96.83	0.010	96.82	96.9	30.087
SVM Kernel	96.69	96.75	0.010	96.82	96.8	878.01
LRK	96.06	96.21	0.012	96.40	96.4	276.76
LD	95.65	95.80	0.014	95.79	95.8	15.669
WNN	96.28	96.34	0.012	96.35	96.4	19.3

Bold represent best values.

**Table 11:** Proposed slicing-based serial feature fusion results on chest X-ray dataset.

**Classifiers**	**Sensitivity**	**Precision**	**FPR**	**F1-score**	**Accuracy**	**Time**
QSVM	98.90	99.23	0.002	99.1	99.4	54.639
CSVM	99.19	99.25	0.002	99.32	99.4	52.913
M G SVM	98.16	98.39	0.005	98.37	98.3	75.546
C G SVM	99.13	99.17	0.002	99.20	99.2	63.507
ESD	99.04	99.07	0.002	99.33	99.3	379.4
LSVM	99.21	99.25	0.002	99.35	99.4	43.302
SVM Kernel	99.26	99.34	0.0019	99.43	99.4	805.69
LRK	99.18	99.26	0.002	99.17	99.2	315.67
LD	98.56	98.65	0.003	98.95	99.0	116.59
**WNN**	**99.63**	**99.79**	**0.002**	**99.38**	**99.4**	**33.657**

Bold represent best values.

### Grad-CAM visualization and comparison

Grad-CAM is a CAM generalization that offers a localization map on the image based on the selected layer. In our work, we utilized global average pooling convolutional (GAP) feature maps that are directly fed into SoftMax (45). Grad-CAM needs to acquire a localization map that discriminates based on social status. Grad-CAM $D_{GRAD-CAM\;}^{c}\in {\mathbb{R}}^{m\times n}$ in deep convolutional neural networks, after a convolutional layer has been trained, its feature mappings β are used to calculate the layer's gradient of *g*^*c*^. Weights $\alpha_{k}^{c}\;$are calculated using global average pooled interpretations of these gradients.


(17) $$\begin{array}{l} \alpha_{k}^{c}=\frac{1}{Z}\underset{i}{\sum }\underset{j}{\sum }\frac{\partial g^{c}}{\partial \beta_{ij}^{k}} \end{array}$$

where weights are represented by $\alpha_{k}^{c}$ that defines the feature map *k* for a specific class *c* and serves as a partial linearization of the deep network downstream of β. It is not necessary for *g*^*c*^ to be a class score; alternatively, it might be anything that can be triggered in a different way. Our Grad-CAM heat map, like CAM, is a weighted combination of feature maps, but we then refine the findings using a ReLU:


(18) $$\begin{array}{l} D_{GRAD-CAM\;}^{c}=RELU\;\left(\underset{k}{\sum }\alpha_{k}^{c}\beta^{k}\right) \end{array}$$

**Figure 11:**
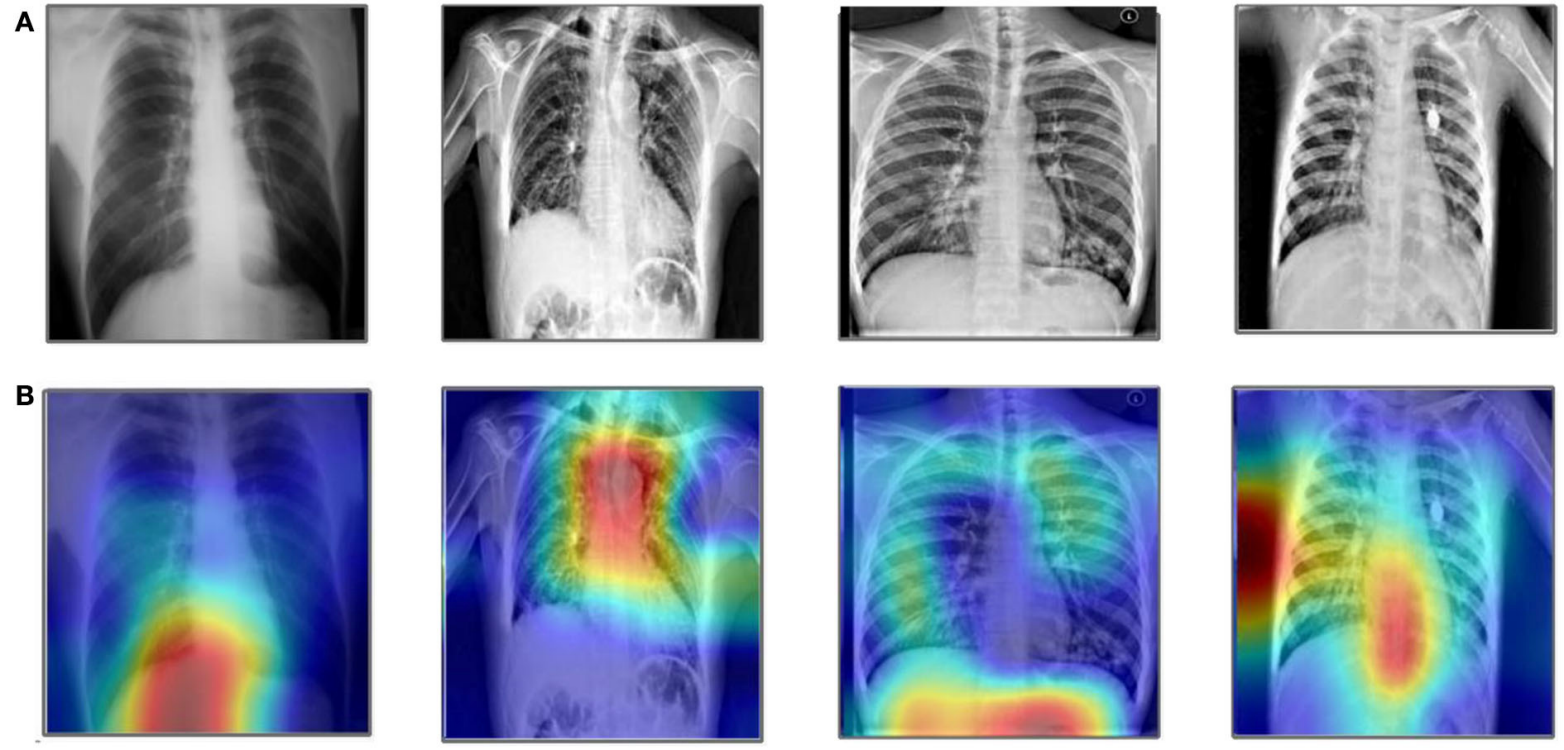
Sample images of Grad-Cam-based analysis. **(A)** Original images and **(B)** Grad-CAM analysis.

This $D_{GRAD-CAM\;}^{c}$ generates the primitive heat map that is normalized for visualization. In our article, we utilized the Grad-CAM for the analysis of the selected models. The Grad-CAM creates a heap map of the infected area of the lungs in the CXR images. A few resultant samples are shown in Figure 11. Finally, Table 12 shows a comprehensive evaluation of many computerized methods. This table contains a large number of newly introduced strategies that all use deep learning and Bayesian optimization concepts. Recently, the maximum accuracy has reached 99.3% by (46). In contrast, the suggested framework obtained a high degree of accuracy, as shown in Table 12. This shows the improvement of the proposed method.

**Table 12:** Comparison of the proposed method to existing techniques.

**Sr. No**	**References**	**Year**	**Method**	**Accuracy (%)**
1	(47)	2020	Detection of COVID19 infection by using deep features and Bayesian optimization	98.97
2	(48)	2022	COVID19 diagnosis using CNN architected and Bayesian Optimization	96.29
3	(46)	2022	Diagnosis of Corona virus on CT images using bayes optimized DCNN and ML	99.3
Proposed	DCNN Bayesian optimization with slicing			98.8
	Serial fusion method			97.9
				**99.4**

Bold represent best values.

## Conclusion

This article presents an automated COVID-19 classification technique based on the hyperparameter optimization of pre-trained deep learning models *via* BO. Initially, contrast is increased to improve the visual quality of the input images, which are later used to train selected pre-trained models. Transfer learning is used to fine-tune and train both models. BO was used to optimize the hyperparameters of selected pre-trained models during training. Following that, features are extracted and fused using a proposed slicing-based approach. Three publicly available datasets were used in the experiment, and the accuracy was higher than with previous techniques. Based on the findings, we concluded that the proposed contrast-enhanced approach improved training capability, allowing for the later extraction of important features. Furthermore, the BO-based hyperparameters selection trained selected models more effectively than static initialization. Furthermore, the proposed fusion method improved classification accuracy. The computational time of the classification accuracy that was increased after the fusion process is the work's limitation. In future, feature selection methods will be prioritized in order to reduce the dimension of fused data.

## Data availability statement

The original contributions presented in the study are included in the article/supplementary material, further inquiries can be directed to the corresponding author/s.

## Author contributions

All authors listed have made a substantial, direct, and intellectual contribution to the work and approved it for publication.

## Funding

This work was supported by the Human Resources Program in Energy Technology of the Korea Institute of Energy Technology Evaluation and Planning (KETEP), granted financial resources from the Ministry of Trade, Industry & Energy, Republic of Korea (No. 20204010600090).

## Conflict of interest

The authors declare that the research was conducted in the absence of any commercial or financial relationships that could be construed as a potential conflict of interest.

## Publisher's note

All claims expressed in this article are solely those of the authors and do not necessarily represent those of their affiliated organizations, or those of the publisher, the editors and the reviewers. Any product that may be evaluated in this article, or claim that may be made by its manufacturer, is not guaranteed or endorsed by the publisher.
